# How do career expectations and job preparation affect youth job satisfaction? An empirical analysis based on underdeveloped regions in China

**DOI:** 10.3389/fpsyg.2026.1847406

**Published:** 2026-06-23

**Authors:** Xin Zhong, ZeKun Wang

**Affiliations:** 1 High-end Think Tank for Local Practice of Modernization of National Governance System and Governance Capacity, Guizhou Academy of Social Sciences, Guiyang, China; 2 School of Public Administration and Law, Hunan Agricultural University, Changsha, China

**Keywords:** career expectations, compensation level, job preparation, job satisfaction, youth in underdeveloped regions

## Abstract

Against the backdrop of intensified labor market competition, youth in underdeveloped regions face heightened structural constraints. It is crucial to understand the specific characteristics of their career expectations and job preparation, as well as the underlying mechanisms influencing their job satisfaction, to support higher-quality employment among this group. This study employs a moderated mediation model, drawing on questionnaire data from 9,627 employed youth aged 18–35 across all prefecture-level cities (prefectures) in G Province, China. Objective salary level (measured by actual monetary income brackets) is incorporated as a mediating variable to explore the pathways linking career expectations and job preparation with overall job satisfaction (defined as the overall subjective evaluation of current employment). The findings indicate that career expectations significantly and positively correlate with job satisfaction, and salary level partially mediates this relationship. Furthermore, job preparation positively moderates the effect of career expectations on salary but negatively moderates their impact on satisfaction. Specifically, better preparation strengthens the translation of career expectations into higher salaries but diminishes the satisfaction gained from career expectations. This phenomenon may stem from the fact that comprehensive job preparation not only elevates actual salary levels but also raises youths’ reference standards regarding career development, social recognition, and related factors, resulting in a comparative gap. Heterogeneity analysis reveals a slightly stronger positive correlation between career expectations and job satisfaction among rural youth compared to urban youth. Moreover, career expectations significantly and positively correlate with salary levels among males, whereas this correlation is not significant among females.

## 1 Introduction

Employment is a fundamental livelihood issue globally, closely tied to workers’ interests and the sustainable development of economic and social systems. Compared to developed regions, youth employment challenges in developing economies and underdeveloped areas are more intricate. These complexities arise from developmental disparities and deep-rooted structural constraints. Firstly, limited industrial diversity and insufficient high-quality jobs lead to a dilemma where available positions often lack optimal conditions and stability (International Labour Organization, 2025; Das, 2025). Secondly, significant employment information asymmetry, coupled with immature employment intermediaries and limited social networks, reduces the compensatory advantage of social capital for youth from rural or low-income backgrounds, further diminishing their labor market competitiveness (Di Giorgio et al., 2024). Thirdly, institutional employment support resources are insufficient, and the public employment service system struggles to meet youths’ diverse needs (Chakravorty et al., 2024). Lastly, a structural mismatch between youths’ accumulated human capital and available positions exists, wherein heightened career expectations generated through education cannot be met by the local availability of high-quality jobs. Thus, the core employment issue lies less in individual abilities and more in systemic mismatches involving employability, career expectations, and high-quality employment opportunities (Belot et al., 2024).

Against this background, youth in underdeveloped regions often confront not only the issue of “whether they can obtain employment,” but more importantly, “whether they can secure jobs that align with their expectations.” Expectancy theory suggests that the intensity of individual motivation depends on a comprehensive evaluation of the effort-performance-outcome relationship. Accordingly, the clearer individuals’ value expectations regarding employment outcomes and the stronger their expectations concerning their ability to attain such outcomes, the more likely they are to devote substantial effort to job searching and selecting positions that correspond to their expectations, thereby promoting consistency between actual work outcomes and subjective goals (Vroom, 1964). The expectancy disconfirmation model (EDM) further proposes that career expectations not only influence pre-employment job selection but also function as a reference standard for evaluating post-job satisfaction (Oliver, 1980). In practice, career expectations shape not only youths’ job selection criteria but also their job satisfaction by influencing their perceptions and evaluations of employment outcomes. Therefore, examining youth employment issues in underdeveloped regions from the perspective of the relationship between career expectations and job satisfaction is of considerable significance.

During the career selection process, job preparation encompasses information acquisition, skill development, and job-search planning, serving as a critical bridge through which youth translate career expectations into actual employment outcomes. In underdeveloped regions, job preparation may influence career expectations through two primary pathways. First, it may enhance job-search skills, information-processing capabilities, and psychological resilience, thereby increasing the likelihood of obtaining desirable employment opportunities. Second, it may reshape individuals’ benchmarks for career expectations through a deeper understanding of occupational environments and salary structures (Marciniak et al., 2020; Chakravorty et al., 2024). Therefore, within the context of underdeveloped regions, the manner in which job preparation interacts with career expectations and salary levels to influence job satisfaction remains insufficiently explored empirically. This gap constitutes the primary rationale for incorporating job preparation into the present analysis and examining its moderating role.

Social Cognitive Career Theory (SCCT) conceptualizes career expectations as “outcome expectations,” whereas job preparation reflects the behavioral manifestation of self-efficacy. Their interaction influences job satisfaction through the mediating role of objective rewards, such as salary level (Lent et al., 1994). Building upon these theoretical foundations, this study develops a multilevel mechanism model linking “subjective cognition (career expectations) → objective reward (salary level) → subjective evaluation (job satisfaction)” and introduces job preparation as a moderating variable. The aim is to reveal the differentiated pathways underlying youth job satisfaction in underdeveloped regions and to provide references for optimizing youth employment support policies and promoting high-quality full employment.

This study offers three primary contributions. First, based on questionnaire data collected from G Province, a representative underdeveloped region, the study objectively captures youths’ subjective evaluations of employment quality in such contexts. Second, it simultaneously examines pre-employment career expectations and job preparation, together with post-employment salary levels, thereby integrating subjective cognition, behavioral preparation, and objective rewards into a unified analytical framework and broadening the research perspective. Third, by focusing on the impact of career expectations on job satisfaction, the study investigates the roles of job preparation and salary level while conducting heterogeneity analyses across urban–rural and gender groups, thereby systematically clarifying the mechanisms underlying youth job satisfaction.

## 2 Literature review

Job satisfaction refers to workers’ evaluations of their current employment or job-related experiences. It represents an affective domain response and is a critical indicator for assessing employment quality at both individual and societal levels (Locke, 1976). Existing studies primarily approach job satisfaction from two perspectives. The first involves individual factors, such as personal cultural background, education level, employment expectations, and intrinsic motivations including personal beliefs, achievement, and responsibility (Dinda, 2024). The second perspective emphasizes environmental factors, such as salary, organizational climate, organizational justice, and job content (Wibowo et al., 2024). Numerous studies have examined career expectations as significant predictors of youth career attainment and indicators of intergenerational mobility, consistently demonstrating that job satisfaction increases significantly when career expectations closely match actual work outcomes (Schmitt-Wilson and Walker, 2016). Some researchers have jointly analyzed educational adaptation and career expectations as determinants of job satisfaction, finding that regardless of educational adaptation, satisfaction remains high when career expectations are met (Song and Zhao, 2022). Other studies explore youth employment opportunities and outcomes from macro- and micro-level perspectives, including economic development, industrial structure, educational investment, and family background. They argue that employment outcomes for youth are jointly shaped by labor market conditions, institutional contexts, and individual resource endowments (Fabrizi and Rocca, 2024). Further studies focusing on youth employment quality highlight position-job matching, salary rewards, and career development opportunities as key dimensions for evaluating high-quality employment (Belot et al., 2024; Chen et al., 2025). Additionally, research on youth occupational mobility contends that mobility not only reflects youths’ adaptability in the labor market (Yang and Hu, 2023) but also their employment quality and job satisfaction. Collectively, these studies deepen our understanding of the “expectation-outcome matching” mechanism.

Nevertheless, existing literature presents at least two unresolved controversies. First, no consensus has been reached regarding the mediating mechanisms linking career expectations and job satisfaction. Most empirical studies grounded in SCCT focus predominantly on outcomes such as occupational interests and career choices. Consequently, there is limited systematic inquiry into whether objective salary levels constitute a distinct mediating pathway or whether differential group performance exists within this pathway (Lent et al., 1994; Zheng et al., 2025). Second, and more fundamentally contentious, the EDM argues that dissatisfaction significantly intensifies when available job positions’ salary levels and development opportunities consistently fall short of optimal reference expectations (Oliver, 1980; Schiebler et al., 2026). For youth in underdeveloped regions, identifying the transition from “expectation confirmation” to “expectation disconfirmation” constitutes the core issue that this study aims to clarify.

In addition, scholars have explored employment status differences, work intensity, and labor remuneration in the employment process. For instance, some argue that increasing wages is more beneficial for improving job satisfaction among young workers outside established institutional employment systems, while reducing overtime better enhances satisfaction among those within these systems. Job satisfaction among youth employed within institutional establishments tends to be higher than among those outside; however, there is a marked unidirectional occupational flow, with significantly more individuals transitioning from within institutional establishments to outside employment than vice versa (Wang and Fang, 2022; Xu and Qi, 2016). Methodologically, existing research utilizes data sources such as the Chinese General Social Survey (CGSS) and questionnaire scales, employing analytical techniques including mediation models, logistic regression, and one-way analysis of variance to examine youth job satisfaction (Xu, 2024; Hong et al., 2023).

Finally, the strength of the relationship between salary level and job satisfaction remains an area of ongoing debate. A meta-analysis of 115 independent samples conducted by Judge et al. (2010) revealed that both objective salary level and salary satisfaction correlate with job satisfaction, but the correlations are relatively weak. This finding implies that the explanatory power of salary for job satisfaction is considerably lower than intuitively expected, suggesting that other factors account for a substantial proportion of the variance in job satisfaction. Under different economic development conditions and welfare system arrangements, the strength of this relationship may exhibit systematic variation. Accordingly, the present study positions salary level as both a mediating and conditional variable, specifically addressing the aforementioned controversies.

Existing studies have provided a valuable foundation for understanding the impact of structural factors on youth job satisfaction, yet several limitations remain. First, most existing research utilizes national-level samples or focuses on developed regions, rarely addressing systematic differences in labor market dynamics, job availability structures, and human capital returns in underdeveloped regions. Second, existing studies typically examine objective employment conditions and subjective factors separately, lacking a unified analytical framework to elucidate their interactions and mediating mechanisms. Particularly, theoretical insights and empirical evidence remain limited regarding how career expectations influence job satisfaction and the role job preparation plays under structural constraints specific to underdeveloped regions. Third, the analytical frameworks of most studies center around observable job characteristics, starting from the question of “whether youth achieve high-quality employment,” while consideration of pre-employment subjective factors, such as career expectations, is typically narrow. Consequently, existing literature struggles to answer a core question: Why do youth with similar salaries and positions exhibit significantly different levels of job satisfaction? This indicates that explanations based solely on objective conditions have reached their explanatory limits, necessitating the incorporation of subjective benchmarks to address the residual variance in satisfaction. Fourth, in underdeveloped regions, the longstanding interplay between urban–rural dual structures and gender-based occupational segregation, coupled with household registration status and traditional gender norms, may reinforce a “core-periphery” division, thereby affecting job satisfaction. Existing knowledge alone cannot clarify the direction or strength of mechanisms linking career expectations and satisfaction across different groups. Empirical cross-group comparisons using identical samples and models are required to provide robust evidence.

In summary, this study empirically examines the employment status and employment quality of youth in underdeveloped regions from the perspective of job satisfaction. Compared to research that exclusively focuses on employment outcomes or objective work conditions, this study integrates pre-employment subjective cognition, behavioral preparation, and post-employment objective rewards into a unified analytical framework. It systematically investigates their impacts on job satisfaction and examines group heterogeneity along urban–rural and gender dimensions, thereby comprehensively revealing the pathways and moderating processes underpinning youth job satisfaction in underdeveloped regions.

## 3 Theoretical basis and research hypotheses

### 3.1 Career expectations and job satisfaction

Expectancy theory argues that an individual’s motivational intensity depends on their comprehensive assessment of whether effort leads to performance, whether performance translates into outcomes, and whether such outcomes are valuable (Vroom, 1964; Huang et al., 2025). In other words, significant motivation emerges only when individuals believe their effort can produce valuable results. Regarding youth employment, career expectations reflect individuals’ subjective anticipations formed prior to labor market entry concerning job responsibilities, salary rewards, career development opportunities, and social recognition. Such expectations influence not only youths’ criteria for selecting job positions but also serve as benchmarks for evaluating their employment outcomes (Wang, 2026). Additionally, the EDM suggests that satisfaction arises from comparing actual perceptions with prior expectations. When actual outcomes meet or exceed expectations, positive evaluations are likely to emerge. Conversely, satisfaction declines when actual outcomes fall short of expectations. Thus, for youth employment, career expectations represent not only subjective pre-employment aspirations but also critical evaluative benchmarks used to judge actual job experiences. Clearer career expectations enable youth to make employment decisions aligned with specific goals, thus enhancing job matching and subsequently improving job satisfaction (Oliver, 1980; Jiang and Wang, 2025). Based on these theoretical considerations, the following empirical hypothesis is proposed:

*H1*: Career expectations are significantly and positively associated with job satisfaction. Specifically, the clearer youths’ pre-employment career expectations, the higher their reported job satisfaction after employment.

### 3.2 Mediating role of salary level

Salary level represents the most direct and tangible objective reward obtained by workers during employment and constitutes a crucial dimension for assessing employment outcomes. Existing studies indicate that salary significantly influences individual employment evaluations, although its relationship with job satisfaction does not always follow a straightforward linear pattern (Judge et al., 2010; Gazi et al., 2024). Specifically, for youth in underdeveloped regions, salary reflects not only economic improvement but also the extent to which their labor input is recognized and their career expectations are fulfilled. Theoretically, youth with clearer career expectations are more likely to emphasize positional rewards, developmental opportunities, and resource alignment during employment decisions, thus achieving superior salary outcomes through more proactive job searching and position selection. Furthermore, as an observable and comparable objective benefit, salary directly shapes youths’ overall evaluations of their current jobs. When actual salary levels meet or exceed initial career expectations, youth are more likely to experience higher job satisfaction. Conversely, significantly lower-than-expected salaries may diminish positive evaluations of their employment (Oliver, 1980). Thus, career expectations may influence job satisfaction both directly and indirectly through salary level (Van Eerde and Thierry, 1996). Accordingly, the following empirical hypothesis is proposed:

*H2*: Career expectations not only directly associate with job satisfaction but also indirectly associate with job satisfaction via the mediating role of salary level.

### 3.3 Moderating role of job preparation

Job preparation refers to behavioral investments individuals undertake before entering the labor market, such as gathering information, developing skills, planning job searches, and psychological preparation, to achieve employment objectives (Marciniak et al., 2020). Unlike career expectations, which primarily represent aspirations and beliefs, job preparation emphasizes tangible actions and readiness before employment. Theoretically, job preparation can moderate the relationship between career expectations and job satisfaction through two explanatory pathways. From a positive perspective, according to SCCT, enhanced job preparation increases individuals’ self-efficacy during job searches, including job search skills, ability to identify suitable positions, and information acquisition, thereby improving their capacity to convert career expectations into actual salary outcomes (Lent et al., 1994; Zou et al., 2025). Thus, youth who engage more extensively in job preparation are more likely to secure employment that aligns closely with their skills and expectations. Based on this theoretical perspective, the following empirical hypothesis is proposed:

*H3*: Job preparation moderates the relationships between career expectations, salary level, and job satisfaction.

*H3a*: Job preparation positively moderates the association between career expectations and salary levels.

Conversely, job preparation’s role in moderating the relationship between career expectations and job satisfaction may not be entirely positive. The preparation process itself involves gaining a deeper understanding of labor market realities, including salary structures, working conditions, and promotion opportunities. This enhanced awareness can produce dual effects: on the one hand, it provides a realistic grasp of market conditions (a beneficial effect); on the other hand, it raises youths’ reference standards from vague, optimistic expectations to clearly informed high standards. When labor markets in underdeveloped regions, characterized by limited job availability and constrained career progression opportunities, cannot meet these elevated standards, job satisfaction is likely to decline rather than increase. This outcome suggests a more nuanced moderating effect of job preparation, reflecting its potentially negative influence. Therefore, the following empirical hypothesis is proposed:

*H3b*: Job preparation negatively moderates the relationship between career expectations and job satisfaction. Specifically, more comprehensive job preparation weakens the positive predictive effect of career expectations on job satisfaction.

### 3.4 Heterogeneity by urban–rural status and gender

Does the effect of career expectations on job satisfaction vary according to urban–rural status and gender? Labor market segmentation theory posits that labor markets are not unified and fully competitive; rather, distinct labor groups are segmented into secondary markets characterized by different compensation levels, job stability, and career opportunities, driven by institutional arrangements, resource endowments, social networks, and identity attributes (Bosworth et al., 1996). Consequently, systematic disparities often arise among groups regarding employment opportunities, salary levels, and career advancement prospects. Specifically, urban–rural differences significantly impact youth in terms of educational resources, employment information, social support, and job opportunities, leading to variations in how career expectations, job preparation, and actual satisfaction interrelate. In underdeveloped regions, market-oriented high-quality positions are limited, and rural youth face greater difficulties than urban youth in translating career expectations into satisfactory employment outcomes. Therefore, salary rewards may operate differently within the relationship between career expectations and job satisfaction (Li and Li, 2022). Similarly, gender differences profoundly shape youth employment experiences. Influenced by occupational gender segregation, income disparities, and unequal promotion opportunities (Anker, 1997; He et al., 2020), male and female youth may differ substantially in the realization of career expectations, returns on job preparation, and the formation of job satisfaction (Clark, 1997). He et al. (2020) found that Chinese women earn approximately 60% of men’s incomes, with the gap widening as income quantiles rise. Gender-based occupational segregation and wage disparities likely make it more challenging for female youth to translate career expectations into salary gains. However, the “paradox of the contented female worker,” as proposed by Clark (1997), suggests that women may maintain relatively high job satisfaction despite lower salaries, implying that female youth might adjust their expectations through adaptive reference standards or reference group selection. Therefore, female youth may compensate for disadvantages in career advancement through enhanced job preparation, particularly if salary mediation effects are insignificant. Rural youth, confronting similarly intensified structural constraints regarding employment opportunities, job quality, and salaries, may face comparable choices. Based on these arguments, the following empirical hypothesis is proposed:

*H4*: The association between career expectations and job satisfaction significantly differs by urban-rural status and gender.

**Figure 1:**
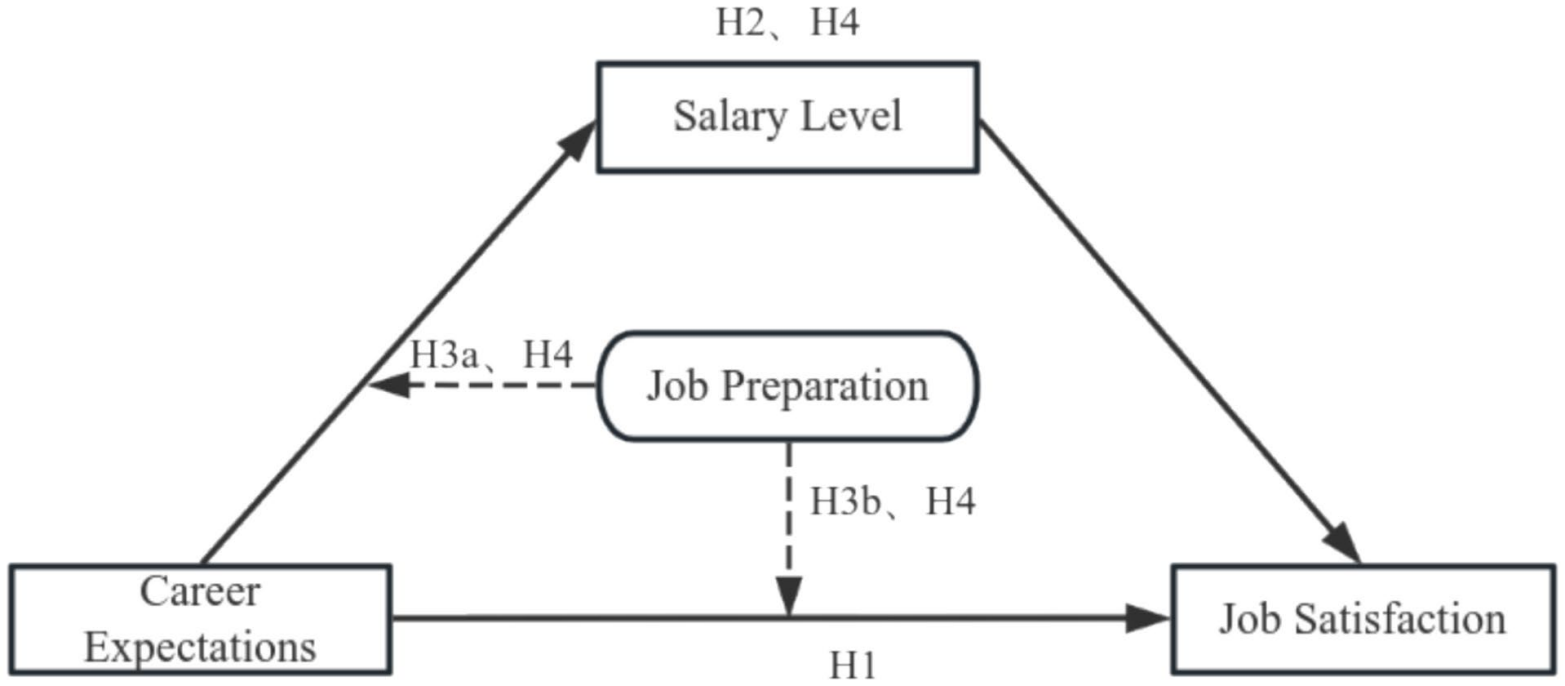
Hypothetical model of moderated mediation effect.

According to the above hypotheses, the following moderated mediation model is proposed (Figure 1).

## 4 Research design

### 4.1 Data source and sample selection

Data used in this study originate from a youth employment status survey conducted in G Province, China. G Province features extensive rural areas, a substantial low-income population, and a per capita net income consistently below the national average, making it representative of underdeveloped regions. The survey employed classic sampling methods (Kish, 1997; Ahmed, 2024) with multi-stage stratified probability sampling. Initially, all prefecture-level cities and autonomous prefectures in G Province were selected as primary sampling units, covering all prefecture-level administrative regions to achieve comprehensive geographic representation and prevent information omission. Subsequently, districts/counties within cities and prefectures were sampled proportionally to their population size. Finally, employed youth aged 16–35 working in both public and private sectors were systematically randomly selected for questionnaire surveys. The number of respondents per region ranged from 934 (6.42%) to 3,119 (21.43%). In total, 10,557 questionnaires were collected, of which 9,627 were valid, yielding an effective response rate of 91.19%. Detailed descriptive statistics of the sample are presented in Table 1.

**Table 1:** Descriptive statistical analysis of the sample.

Variable	Category	Count	Proportion
Age	18 years old and below	68	0.70%
	19–22 years old	790	8.20%
	23–29 years old	6,148	63.90%
	30–35 years old	2,621	27.20%
Gender	Male	3,442	35.80%
	Female	6,185	64.20%
Education	High school and below (including secondary vocational school)	780	8.10%
	Junior college	2,315	24.00%
	Bachelor’s degree	6,359	66.10%
	Master’s degree	167	1.70%
	Doctoral degree	6	<0.1%
Urban–rural distribution	Urban household registration	2,711	28.20%
	Rural household registration	6,916	71.80%

### 4.2 Model specification

This study employs multiple linear regression analysis to investigate the effects of career expectations on salary levels and job satisfaction among youth in underdeveloped regions. To examine how career expectations influence job satisfaction, the PROCESS plugin is utilized to test the mediating effect of salary level and the moderating effect of job preparation.

#### 4.2.1 Baseline regression model


$$Y_{\mathrm{ij}}=\alpha_{0}+\alpha_{1}X_{\mathrm{ij}}+\alpha_{2}C_{\mathrm{ij}}+\varphi_{\mathrm{ij}}$$ (1)

Equation 1 represents the direct association between career expectations and job satisfaction. Where $Y_{\mathrm{ij}}$ is the dependent variable (job satisfaction); $X_{\mathrm{ij}}$ is the independent variable (career expectations); $\phi_{\mathrm{ij}}$ is the residual term. $\alpha_{0}$ is the constant term; $\alpha_{1}$ reflects the effect of career expectations on job satisfaction; $\alpha_{2}$ is the regression coefficients of the control variables.

#### 4.2.2 Mediation effect model

To examine the mediating effect of salary level, a mediation model is constructed based on existing literature (Wen and Ye, 2014; Preacher and Hayes, 2008):


$$M_{\mathrm{ij}}=\beta_{0}+\beta_{1}X_{\mathrm{ij}}+\beta_{2}C_{\mathrm{ij}}+\varepsilon_{\mathrm{ij}}$$ (2)

$$Y_{\mathrm{ij}}=\gamma_{0}+\gamma_{1}X_{\mathrm{ij}}+\gamma_{2}M_{\mathrm{ij}}+\gamma_{3}C_{\mathrm{ij}}+\eta_{\mathrm{ij}}$$ (3)

Equations 2 and 3 test the mediation effect of salary level. Where $M_{\mathrm{ij}}$ represents the mediating variable (salary level); $\beta_{0}$ and $\gamma_{0}$ are constant terms, and the meanings of $\beta_{1}$ , $\gamma_{1}$ , and $\alpha_{1}$ are the same.

#### 4.2.3 Moderated mediation effect model

Building upon the above models, the moderated mediation effect model is further developed as follows:


$$M_{\mathrm{ij}}=\lambda_{0}+\lambda_{1}X_{\mathrm{ij}}+\lambda_{2}X_{\mathrm{ij}}*W_{\mathrm{ij}}+\lambda_{3}C_{\mathrm{ij}}+\mu_{\mathrm{ij}}$$ (4)

$$Y_{\mathrm{ij}}=\theta_{0}+\theta_{1}X_{\mathrm{ij}}+\theta_{2}W_{\mathrm{ij}}+\theta_{3}X_{\mathrm{ij}}*W_{\mathrm{ij}}+\theta_{4}M_{\mathrm{ij}}+\theta_{5}C_{\mathrm{ij}}+\xi_{\mathrm{ij}}$$ (5)

Equations 4 and 5 examine the moderated mediation effects, where $W_{\mathrm{ij}}$ is the moderating variable (job preparation); $X_{\mathrm{ij}}*W_{\mathrm{ij}}$ is the interaction between career expectations and job preparation. The constant terms are $\lambda_{0}$ and $\theta_{0}$ , and $\lambda_{1}$ – $\lambda_{3}$ , $\theta_{1}$ – $\theta_{4}$ represent the regression coefficients.

It should be noted that this study does not employ a mediated moderation model, as such a model presupposes that moderation initially influences the direct relationship between independent and dependent variables, with the mediating variable subsequently transmitting this moderating effect. The central focus of this study is whether the mediating pathway (salary level mediating the relationship between career expectations and job satisfaction) is moderated by job preparation, rather than if the moderation itself is mediated. Therefore, a moderated mediation model aligns appropriately with the research objectives.

### 4.3 Variable definition

#### 4.3.1 Explained variable: job satisfaction

In this study, job satisfaction is operationalized as employed youths’ overall subjective evaluation of their current employment. A meta-analysis by Wanous et al. (1997) involving 7,682 participants demonstrated adequate convergent validity for single-item measures. Cheung and Lucas (2014) similarly argued that single-item measures effectively capture individual job satisfaction in social surveys, showing strong consistency with multi-item scales across three large-scale samples. Additionally, Pelly (2023) validated single-item job satisfaction measures as practical. Adhering to these methodological guidelines, which effectively reduce respondent burden and increase response rates during surveys, this study uses a single-item measure: “Are you satisfied with your current job?” Responses include five categories: “Very dissatisfied,” “Somewhat dissatisfied,” “Neutral,” “Satisfied,” and “Very satisfied,” scored from 1 to 5, respectively.

#### 4.3.2 Explanatory variable: career expectations

Career expectations are operationalized as youths’ cognitive schemas and multidimensional evaluations of ideal jobs prior to entering the labor market. According to expectancy theory, prospective employees select careers based on their needs, with higher valence and stronger expectations for goals (e.g., location, interests, social status) resulting in stronger motivational forces. Reflecting contemporary demands, the vocational interest scale developed by Wang et al. (2024) highlights the emerging significance of “work freedom” and “development opportunities” among youth. Integrating relevant items from the National College Graduate Employment Status Questionnaire, this study constructs a career expectation measurement framework encompassing six core dimensions, balancing “spiritual adaptation” and “material adaptation”: (1) Work location (geographical preferences); (2) Social status (prestige); (3) Interests and hobbies (intrinsic value); (4) Career development prospects (long-term growth expectations); (5) Equal development opportunities (perceived fairness); (6) Work freedom (new demands in the digital era). Items are presented as: “Before looking for this job, you paid more attention to. Do you agree?” Responses include five categories: “Strongly disagree,” “Somewhat disagree,” “Neutral,” “Agree,” and “Strongly agree,” scored from 1 to 5, respectively.

#### 4.3.3 Mediating variable: salary level

In this study, salary level is operationalized as the objective monthly income of employed youth. The Pay Satisfaction Questionnaire, developed by Heneman and Schwab (1985), distinguishes four dimensions, pay level, raises, benefits, and pay structure/administration, and was among the first instruments to emphasize employees’ evaluations of actual salary amounts in the pay-level dimension. The current study incorporates a 5-point ordinal salary scale into the regression analysis as a continuous variable, primarily following simulation research by Rhemtulla et al. (2012), who systematically compared the performance of robust maximum likelihood estimation and categorical least squares methods when ordinal variables are treated as continuous. Their findings demonstrated minimal bias and good statistical properties for ordinal variables with five or more categories treated continuously. Therefore, this study utilizes an objective bracket measurement by asking respondents: “Which of the following brackets does your current monthly income fall into?” Responses include five categories: “≤3,000 yuan = 1,” “3,001–4,500 yuan = 2,” “4,501–6,000 yuan = 3,” “6,001–7,500 yuan = 4,” and “≥7,501 yuan = 5.” The choice of income brackets was guided by three main considerations: first, bracket measures reduce respondents’ sensitivity to disclosing exact income and enhance response rates; second, bracket data effectively reflect hierarchical salary differences, facilitating subsequent regression analysis; third, youth income in underdeveloped regions is typically concentrated within these brackets, which adequately capture their salary distribution.

#### 4.3.4 Moderating variable: job preparation

Job preparation refers to the series of behavioral investments youth undertake before entering the labor market to achieve employment goals. Given that job preparation is not a standalone concept, this study draws from scales and prior research identifying and evaluating 12 key dimensions of job preparation (Lent et al., 1994; Marciniak et al., 2020). Combining these findings with the specific context of youth employment in underdeveloped regions, the current study establishes measurement items across five dimensions: (1) Self-cognition (degree of awareness regarding one’s career interests, abilities, and values); (2) Career goals (clarity and specificity of career objectives); (3) Work (internship) experience (extent of accumulated practical experience); (4) Employment policies (knowledge of labor market policies and information); and (5) Job search skills (practical competencies including resume writing, interview preparation, and effective use of job search channels). Items for each dimension follow a standardized question format: “In your pre-employment preparation, your… was very…, do you agree?,” designed to mitigate social desirability bias. Responses range across five categories: “Strongly disagree,” “Somewhat disagree,” “Neutral,” “Agree,” and “Strongly agree,” assigned numerical values from 1 to 5, respectively.

#### 4.3.5 Control variables

To minimize omitted-variable bias, this study incorporates three categories of control variables into the regression analysis. First, basic socio-demographic characteristics include gender (an essential moderator influencing labor market satisfaction), age (significant differences exist in job satisfaction at various career stages), and urban–rural differences (household registration and regional development gaps result in persistent disparities in employment information and social capital, constituting a key structural factor shaping job satisfaction). Second, the study controls for human capital endowment, specifically educational level. Although the meta-analysis by Ng and Feldman (2009) found the overall correlation between education and job satisfaction close to zero, education’s influence operates through two opposing pathways: enhancing work-related resources and elevating expectations. Thus, controlling education remains necessary. Third, perceptions of the employment situation are controlled. According to the EDM, individuals perceiving the employment environment as harsh tend to lower reference expectations, making actual outcomes more likely to surpass expectations and thereby improving satisfaction. Conversely, perceptions of leniency elevate reference standards, complicating satisfaction attainment. Without controlling this variable, perceived macro-level difficulty could become a confounding factor: youth with higher career expectations might be inherently more optimistic, indirectly affecting satisfaction via perceptions rather than actual salary levels. Controlling for employment situation perceptions effectively excludes this alternative explanatory pathway.

## 5 Empirical analysis

### 5.1 Reliability and validity analysis

This study assesses scale reliability using Cronbach’s *α* coefficient. Reliability tests conducted on each latent variable indicate that Cronbach’s α values exceed the acceptable threshold of 0.7, confirming strong internal consistency and suitability for subsequent empirical analysis. As shown in Table 2, the composite reliability (CR) values of all variables surpass the recommended standard of 0.7 (Hair et al., 2010), indicating satisfactory reliability. Additionally, factor loadings for all scale indicators exceed 0.7, demonstrating strong convergent validity. Furthermore, average variance extracted (AVE) values significantly exceed the standard threshold of 0.5. Bartlett’s sphericity test yielded significant results (*p* < 0.01), and the Kaiser-Meyer-Olkin (KMO) value approaches 0.9, reflecting robust partial correlations among variables and further confirming the appropriateness of factor analysis. The measurement items consistently exhibit maximum loadings (>0.6) on their corresponding factors without cross-loading, suggesting strong discriminant validity. Collectively, these results indicate that the dataset is suitable for factor analysis.

**Table 2:** Reliability and validity analysis.

Variable	Indicator	Standard factor loading	Cronbach’s α	CR	AVE	KMO and bartlett test of sphericity
Job preparation	W1-Self-cognition	0.81	0.915	0.751	0.938	KMO = 0.842 Bartlett χ^2^ = 36994.909 Sig. = 0.000
	W2-Career goal	0.872				
	W3-Work (internship) experience	0.861				
	W4-Employment policy	0.898				
	W5-Job search skills	0.89				
Career expectations	X1-Work location	0.806	0.914	0.706	0.935	KMO = 0.901 Bartlett χ^2^ = 40344.019 Sig. = 0.000
	X2-Social status	0.729				
	X3-Interests	0.853				
	X4-Career development prospects	0.892				
	X5-Equal development opportunities	0.895				
	X6-Work freedom	0.856				

Specifically, Cronbach’s α for the career expectations scale (6 items) is 0.914, and for the job preparation scale (5 items) is 0.915, both far exceeding the 0.70 threshold, demonstrating excellent internal consistency. Job satisfaction is measured with a single item; thus, internal consistency measures are inapplicable. However, previous meta-analyses (Wanous et al., 1997) confirmed high correlations between single-item and multi-item satisfaction measures. In this study, correlations between job satisfaction and job preparation (*r* = 0.636, *p* < 0.01), career expectations (*r* = 0.479, *p* < 0.01), and salary level (*r* = 0.183, *p* < 0.01) align closely with theoretical expectations, providing additional criterion-related validity evidence.

### 5.2 Common method bias test

Initially, the traditional Harman single-factor test was employed to assess common method bias. Exploratory factor analysis (principal component analysis with maximum variance orthogonal rotation) yielded no single dominant common factor, indicating no severe common method bias. Specifically, one factor emerged from the career expectations scale, explaining 70.63% of total variance, with all factor loadings exceeding 0.70. Similarly, one factor was extracted from the job preparation scale, accounting for 75.11% of total variance, again with factor loadings exceeding 0.70.

Confirmatory factor analysis (CFA) using Mplus 8.3 software with the WLSMV estimation method provided further verification (Table 3). The results indicate that the revised two-factor model fits the data well, whereas the single-factor model showed substantially poorer fit, confirming that measurement items cannot be fully attributed to a single common factor and thus alleviating concerns regarding common method bias. Additionally, the unmeasured latent method construct (ULMC) analysis showed the method factor’s variance was not significant (variance = 0.038, *p* = 0.293), and standardized factor loadings changed minimally (0.003–0.012), well below the threshold of 0.20 recommended by Podsakoff et al. (2003) and Pereyra-Rojas et al. (2017). Thus, common method variance does not substantively impact the measurement model. Finally, variance inflation factor (VIF) values range between 1.024 and 2.235, all below the conservative threshold of 3.3 (Kock, 2015), indicating no significant multicollinearity concerns.

**Table 3:** Common method bias diagnosis based on CFA and ULMC.

Model	χ^2^	df	χ^2^/df	CFI	TLI	RMSEA	90% CI for RMSEA	SRMR
Modified two-factor model	2304.951	39	59.101	0.992	0.989	0.078	0.075–0.080	0.018
Single-factor model	31603.911	44	718.271	0.894	0.867	0.273	0.270–0.275	0.106
ULMC model	2469.989	38	64.999	0.992	0.988	0.082	0.079–0.084	0.018

*χ*^2^ = Chi-square; df = degrees of freedom; CFI = comparative fit index; TLI = Tucker–Lewis index; RMSEA = root mean square error of approximation; SRMR = standardized root mean square residual; ULMC = unmeasured latent method construct. The method-factor variance was not significant (variance = 0.038, *p* = 0.293). Changes in standardized factor loadings after adding the method factor ranged from 0.003 to 0.012.

### 5.3 Descriptive statistics and correlation analysis

Table 4 presents the means (M), standard deviations (SD), reliability coefficients (Cronbach’s *α*), and bivariate correlation coefficients for each variable. Career expectations (M = 0.00, SD = 1.00) and job preparation (M = 0.00, SD = 1.00) are standardized factor scores with distributions centered at zero and a standard deviation of one. The average job satisfaction score is 3.56 (SD = 0.91), indicating a moderate-to-high satisfaction level on the five-point scale. The average salary level is 1.90 (SD = 0.90), showing that the monthly income of youth in underdeveloped regions typically ranges between ≤3,000 yuan and 4,500 yuan.

**Table 4:** Correlation analysis among study variables.

Variable	Gender	Age	Urban–rural difference	Education	Perception of employment situation	Compensation level	Career expectations	Job preparation	Job satisfaction
Gender	—								
Age	−0.044**	—							
Urban–rural difference	0.004	−0.114**	—						
Education	0.069**	0.033**	−0.086**	—					
perception of employment situation	−0.061**	0.135**	−0.022*	−0.116**	—				
Compensation level	−0.105**	0.232**	−0.129**	0.231**	0.094**	—			
Career expectations	−0.041**	0.076**	−0.059**	0.047**	0.389**	0.094**	—		
Job preparation	−0.073**	0.126**	−0.064**	0.006	0.559**	0.141**	0.567**	—	
Job satisfaction	−0.055**	0.132**	−0.066**	−0.015	0.652**	0.183**	0.499**	0.636**	—
Mean	1.64	3.18	1.72	2.62	3.24	1.9	0	0	3.56
Standard deviation	0.479	0.593	0.45	0.661	1.023	0.901	1	1	0.905
Cronbach‘s α	—	—	—	—	—	—	0.914	0.915	—

*p* < 0.05, **p < 0.01 (two-tailed test). Gender coding: 0 = male, 1 = female; Urban–rural coding: 0 = urban, 1 = rural. Age coding: ≤18 years = 1, 19–22 years = 2, 23–29 years = 3, 30–35 years = 4. Education coding: High school or below = 1, Junior college = 2, Bachelor’s degree = 3, Master’s degree = 4, Doctoral degree = 5. Career expectations and job preparation are standardized factor scores (M = 0, SD = 1). Job satisfaction is measured by a single item; thus, Cronbach’s α is not applicable.

Correlation analyses indicate significant positive correlations between career expectations and job satisfaction (*r* = 0.479, *p* < 0.01), job preparation and job satisfaction (*r* = 0.640, *p* < 0.01), and salary level and job satisfaction (*r* = 0.183, *p* < 0.01), aligning with theoretical predictions. The correlation between career expectations and salary level is relatively weak (*r* = 0.094, *p* < 0.01), whereas a moderate positive correlation exists between job preparation and career expectations (*r* = 0.567, *p* < 0.01).

### 5.4 Baseline effects test

The baseline regression results (Table 5) indicate strong model fit (*R* = 0.703, *R*^2^ = 0.492), with independent variables explaining 49.35% of variance in job satisfaction (*F* = 1561.891, *p* < 0.0001). The regression coefficient for career expectations on job satisfaction (*β* = 0.213, *p* < 0.001) demonstrates a significant positive association, supporting Hypothesis 1. Overall, the baseline regression provides a robust foundation for further mediation and moderation analyses.

**Table 5:** Baseline effects model: career expectations on job satisfaction.

Variable	B	SE	*t*	95% CI	
				LLCI	ULCI
Intercept	1.8845***	0.0628	30.0186	1.7614	2.0075
Career expectation	0.2463***	0.0072	33.9927	0.2321	0.2605
Gender	−0.02	0.0138	−1.4532	−0.047	0.007
Age	0.0519***	0.0113	4.606	0.0298	0.074
Urban–rural difference	−0.0653***	0.0148	−4.4243	−0.0943	−0.0364
Education	0.0435***	0.0101	4.3028	0.0237	0.0634
Perception of employment situation	0.4763***	0.0072	66.4315	0.4623	0.4904
*R*	0.703				
*R* ^2^	0.494				
*F*	1561.891***				

*p < 0.1, **p < 0.05, ***p < 0.01.

### 5.5 Mediation model test

The mediation model was tested using the PROCESS program developed by Hayes (2013), to assess whether salary level mediated the relationship between career expectations and job satisfaction, after controlling for gender, age, urban–rural distribution, education level, and perception of employment situation. After accounting for these variables (Table 6), career expectations were significantly positively correlated with job satisfaction (*b* = 0.246, *p* < 0.001) and with salary level (*b* = 0.028, *p* < 0.005). Salary level was also significantly positively correlated with job satisfaction (*b* = 0.098, *p* < 0.001). When salary level was added as a mediator, the direct relationship between career expectations and job satisfaction remained significant (*b* = 0.244, *p* < 0.001). This indicates that career expectations and salary level positively contribute to job satisfaction.

**Table 6:** Analysis of the mediating effect of salary level between career expectations and job satisfaction.

Variable	Dependent variable: job satisfaction			Dependent variable: salary level			Dependent variable: job satisfaction		
	*b*	SE	*t*	*b*	SE	*t*	*b*	SE	*t*
Gender	−0.020	0.014	−1.453	−0.199***	0.018	−11.088	−0.0004	0.014	−0.031
Age	0.052***	0.011	4.606	0.301***	0.015	20.454	0.022*	0.011	1.956
Urban–rural difference	−0.065***	0.015	−4.424	−0.165***	0.019	−8.551	−0.049***	0.015	−3.342
Education	0.044***	0.010	4.303	0.316***	0.013	23.903	0.013	0.010	1.121
Perception of employment situation	0.476***	0.007	66.431	0.065***	0.009	6.926	0.470***	0.007	65.923
Career expectation	0.246***	0.007	33.992	0.028**	0.010	3.008	0.244***	0.007	33.869
Salary level							0.098***	0.008	12.690
*R* ^2^	0.494			0.131			0.502		
*F*	1561.891***			241.611***			1384.036***		

*p < 0.1, **p < 0.05, ***p < 0.01.

The association between career expectations and salary level was significant (*B* = 0.0284, *t* = 3.008, 95% CI [0.0099, 0.0470], *p* < 0.01). Likewise, the total effect of career expectations on job satisfaction was significant (*B* = 0.2463, *t* = 33.993, 95% CI [0.2321, 0.2605], *p* < 0.01). These findings indicate that clearer career expectations positively correlate with job satisfaction, supporting Hypothesis H1.

After introducing the mediator (salary level), the direct effect of career expectations on job satisfaction remained significant (*B* = 0.2435, *t* = 33.869, 95% CI [0.2294, 0.2576], *p* < 0.01). Additionally, the mediating effect of salary level was significant (indirect effect = 0.0028, Bootstrap 95% CI [0.0008, 0.0047]), accounting for 1.14% of the total effect (Table 7). This result shows that career expectations influence job satisfaction directly and indirectly through salary level, confirming the partial mediation and supporting Hypothesis H2.

**Table 7:** Decomposition of mediating effect of salary level.

Effect	B	SE	95% CI		Percentage
			LLCI	ULCI	
Total effect	0.2463	0.0072	0.2321	0.2605	100%
Direct effect	0.2435	0.0072	0.2294	0.2576	98.86%
Indirect effect(s)	0.0028	0.0010	0.0008	0.0047	1.14%

X = career expectations; M = salary level; Y = job satisfaction. Based on 5,000 Bootstrap resamples. Control variables include gender, age, urban–rural difference, education level, and perception of employment situation.

### 5.6 Moderated mediation model test

After confirming the mediating role of salary level, the moderated mediation model was examined using the PROCESS program, controlling for demographic variables. Results showed significant interaction effects between career expectations and job preparation on both salary level (*B* = 0.018, *p* < 0.01) and job satisfaction (*B* = −0.021, *p* < 0.01), suggesting moderation by job preparation (Table 8). This supports Hypothesis H3.

**Table 8:** Moderated mediation model test.

Outcome variable	Regression model	Model fit indices			Coefficient significance			
	Predictor variable	*R*	*R* ^2^	F(df)	B	*t*	95%CI	
Salary level		0.368	0.135	187.770***				
	Gender				−0.190	−10.522***	−0.225	−0.154
	Age				0.296	20.145***	0.267	0.325
	Urban–rural difference				−0.159	−8.239***	−0.196	−0.121
	Education				0.314	23.782***	0.288	0.340
	Perception of employment situation				0.038	3.659***	0.018	0.058
	Career expectation				0.010	0.892	−0.012	0.031
	Job preparation				0.068	5.784***	0.045	0.091
	Career expectation*Job preparation				0.018	3.444***	0.008	0.029
Job satisfaction		0.745	0.555	1329.880***				
	Gender				0.008	0.582	−0.017	0.034
	Age				0.009	0.859	−0.012	0.031
	Urban–rural difference				−0.040	−2.859**	−0.067	−0.013
	Education				0.003	0.275	−0.017	0.022
	Perception of employment situation				0.367	49.356***	0.353	0.382
	Salary level				0.085	11.601***	0.071	0.100
	Career expectation				0.116	14.621***	0.100	0.131
	Job preparation				0.281	33.349***	0.265	0.298
	Career expectation*Job preparation				−0.021	−5.399***	−0.028	−0.013

*p < 0.1, **p < 0.05, ***p < 0.01.

**Figure 2:**
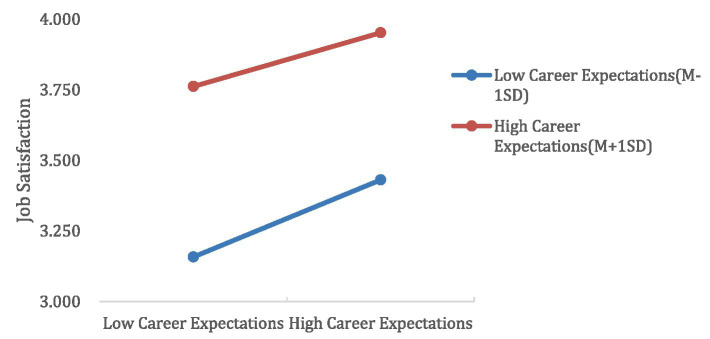
Moderating effect of job preparation on job satisfaction.

Figure 2 demonstrates that job preparation level influences the positive relationship between career expectations and job satisfaction. The slope lines display an upward trend, indicating that as career expectations increase, job satisfaction also rises. However, the slope is steeper at low preparation levels compared to high preparation levels, indicating that increased job preparation weakens this positive correlation. Specifically, the positive effect is strongest at low job preparation levels (effect = 0.136, *p* < 0.01), moderate at average levels (effect = 0.116, *p* < 0.01), and weakest, though still significant, at high levels (effect = 0.010, *p* < 0.01). Johnson–Neyman analysis identified no significant transition point, meaning this moderation effect remains significant throughout the observed range. Thus, job preparation negatively moderates the direct positive effect of career expectations on job satisfaction, confirming the “reference standard upward adjustment” hypothesis. Therefore, H3b is supported with a negative moderating direction.

**Figure 3:**
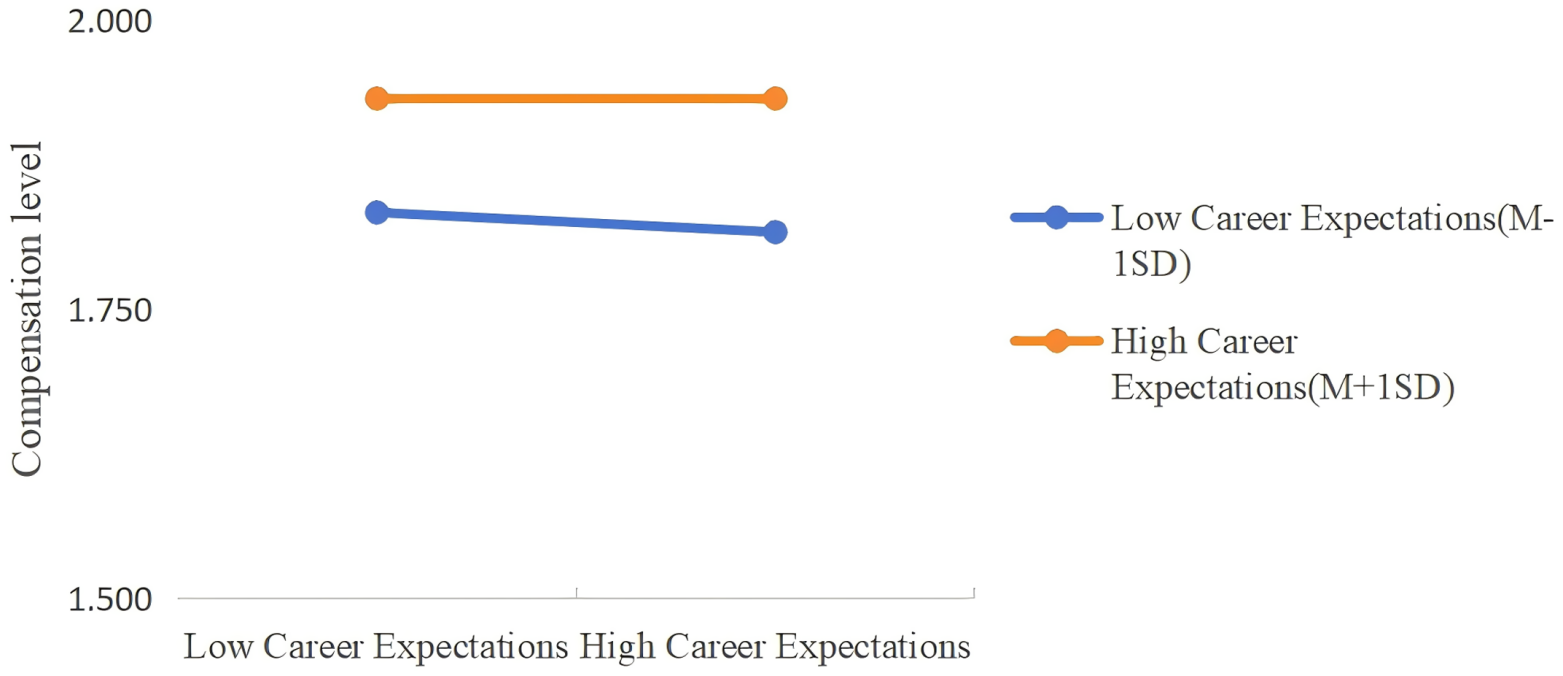
Moderating effect of job preparation on salary level.

Applying the same analytical method, further analysis examined the moderating role of job preparation on the relationship between career expectations and salary level. Results indicated that at low levels of job preparation, the moderating slope was negative; at high preparation levels, the slope was positive. Thus, as job preparation increases, the positive correlation between career expectations and salary level strengthens significantly (*B* = 0.018, SE = 0.005, *t* = 3.444, *p* = 0.0006, 95% CI [0.0079, 0.0287]). Specifically, at low (−1 SD) and average job preparation levels, the correlation between career expectations and salary was not significant (low: effect = −0.009, *p* = 0.437; average: effect = 0.010, *p* = 0.372). At high preparation levels (+1 SD), however, a significant positive correlation emerged (effect = 0.028, *p* = 0.036). Johnson–Neyman significance analysis indicated that career expectations significantly impact salary when job preparation surpasses a standardized value of approximately 0.848. Therefore, job preparation enhances the conversion of career expectations into actual salary, suggesting that better-prepared young individuals are more capable of translating high expectations into higher salaries (Figure 3). Hence, H3a is supported.

**Figure 4:**
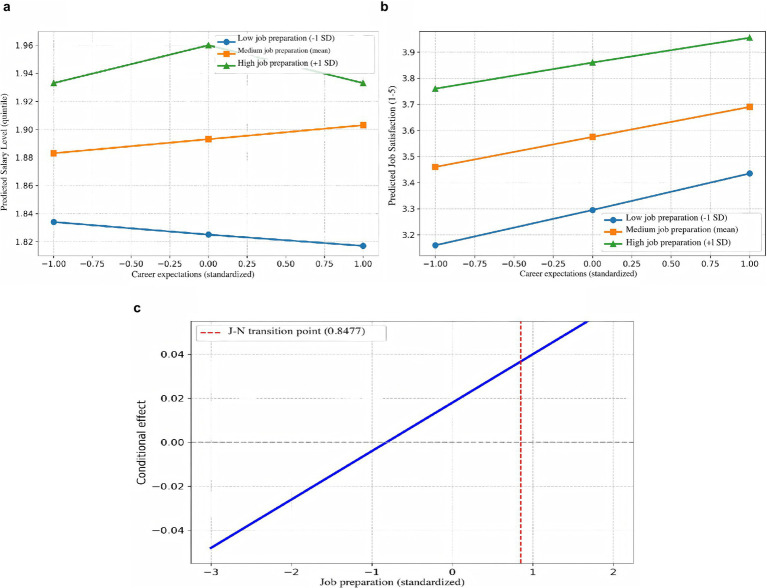
Summary diagram of moderated mediation effects. **(a,c)** Conditional effect of career expectations on salary level as a function of job preparation. **(b)** Moderating effect of job preparation on the direct relationship between career expectations and job satisfaction.

Figure 4 illustrates that job preparation simultaneously serves as an “empowerment” and a “limitation” factor in the relationship between career expectations and job satisfaction. Specifically, Figure 4a indicates that a significant positive correlation between career expectations and salary only emerges at high job preparation levels (+1 SD). At low or average preparation levels, this correlation remains insignificant. Johnson–Neyman analysis (Figure 4c) further shows this positive effect activates only when job preparation exceeds approximately 0.85 in standardized units. Conversely, Figure 4b highlights the negative moderation effect of job preparation on the direct relationship between career expectations and job satisfaction. Although career expectations consistently positively influence satisfaction, this influence weakens as job preparation increases (slope decreases from 0.136 to 0.095). These findings highlight a “preparation paradox”: adequate job preparation facilitates the translation of expectations into higher salaries (positive aspect), but simultaneously diminishes the psychological satisfaction derived from expectations due to the upward adjustment of personal standards (negative aspect). This phenomenon is particularly pronounced under structural constraints in less developed regions.

### 5.7 Robustness tests

#### 5.7.1 Bootstrap test of moderated mediation indicators

The empirical analysis employed the PROCESS macro to further examine whether the moderated mediation model remains valid across different values of the moderating variable. The resulting index value was 0.0016, with a 95% confidence interval (CI) excluding zero, thus confirming the validity of the moderated mediation model proposed in this study.

#### 5.7.2 Test using WLSMV estimation method with the lavaan mediation model

This study initially utilized the PROCESS macro for testing mediation and moderation models, which assumes continuity of the mediator and dependent variables. However, treating five-category ordinal variables as continuous under an OLS framework may introduce estimation bias. Therefore, the mediating variable was incorporated into a mediation model constructed using lavaan, applying the WLSMV estimation method suitable for ordinal categorical data. In this model, career expectations were significantly and positively associated with salary level, and salary level was significantly positively associated with job satisfaction. The total effect of career expectations on job satisfaction was significant. After introducing salary level as the mediating variable, its mediating effect remained significant (*B* = 0.005, *p* = 0.002, 95% CI = [0.002, 0.009]), and the direct effect of career expectations on job satisfaction continued to be significant (*B* = 0.354, *p* < 0.001). The model accounted for 16.7% of the variance in salary level and 50.2% of the variance in job satisfaction. After confirming the significant mediating role of salary level, further analysis controlled for gender, age, urban–rural status, education level, and perception of employment situation. The results showed that, despite minor coefficient variations, the interaction term between career expectations and job preparation significantly and positively correlated with salary level (*B* = 0.042, *p* = 0.001), indicating a significant moderating effect of job preparation on the positive relationship between career expectations and salary level. Simultaneously, this interaction term exhibited a significant negative correlation with job satisfaction (*B* = −0.040, *p* < 0.001), indicating job preparation significantly moderates the correlation between career expectations and job satisfaction. These analyses confirmed that previously obtained conclusions regarding mediation effects, moderated mediation pathways, and related hypothesis tests were robust. The core findings remained consistent after altering the estimation approach, indicating reliability and an absence of estimation bias arising from the treatment of variable measurement scales.

#### 5.7.3 Test based on increasing the number of bootstrap resamples

To verify the stability of parameter estimates, the number of bootstrap resamples for testing mediation and moderated mediation effects was increased from 5,000 to 20,000. Results demonstrated negligible variations in regression coefficients, standard errors, t-values, and significance levels of key variables (differences appeared only at the fourth decimal place and were minimal enough to be ignored). Additionally, the 95% confidence interval boundaries for critical indicators (e.g., Index of Moderated Mediation) changed by less than ±0.001 and consistently excluded zero. Given the substantial sample size (N = 9,627) and the adequacy of the model specification, 5,000 bootstrap resamples proved sufficient for achieving stable parameter estimates. Increasing to 20,000 resamples did not alter any statistical inference outcomes, further confirming the high numerical stability and reproducibility of the study’s findings and the absence of sampling variability bias due to insufficient simulation iterations.

### 5.8 Heterogeneity analysis

#### 5.8.1 Urban–rural heterogeneity analysis

Before the reform, the household registration system functioned similarly to a passport system. On the one hand, it divided the population into agricultural and non-agricultural registrations, assigning the latter higher economic and social status. On the other hand, it restricted spatial and social mobility among the population (Chan and Zhang, 1999). With reforms to the household registration system, direct occupational segregation and differences in socio-economic welfare entitlements based on registration categories have largely disappeared. However, urban–rural disparities remain pronounced, particularly reflected in inequalities concerning economic development, basic education, and cultural life. Such inequalities are especially evident in underdeveloped regions, leading to differences in career choices and development trajectories.

This study employed a multi-group structural equation model to evaluate urban–rural differences in path coefficients. First, an unconstrained model was established, and subsequently, key path coefficients were constrained to equality across groups. The chi-square difference test was then used to assess the significance of these differences. Results indicated no significant urban–rural differences in the relationship between career expectations and salary level (Δχ^2^ = 1.231, *p* > 0.05) or between salary level and job satisfaction (Δχ^2^ = 1.252, p > 0.05); salary level positively and significantly influenced job satisfaction similarly across urban and rural groups. However, a significant urban–rural difference emerged in the relationship between career expectations and job satisfaction (Δχ^2^ = 1082.2, *p* < 0.001), with a stronger correlation observed among rural respondents compared to their urban counterparts. Furthermore, urban–rural differences were not significant in the moderating effects of job preparation on the pathways from career expectations to salary level or from career expectations to job satisfaction (Tables 9, 10).

**Table 9:** Heterogeneity of the mediation model between urban and rural groups.

Path	Rural (*n* = 6,916)	Urban (*n* = 2,711)	Δ*χ*^2^	*p*-value
Career expectation → Salary level	0.020	0.043*	1.231	0.267
Salary level → Job satisfaction	0.092***	0.112***	1.252	0.263
Career expectation → Job satisfaction	0.245***	0.238***	1082.2***	<0.001

*p < 0.1, **p < 0.05, ***p < 0.01.

**Table 10:** Heterogeneity of the moderated mediation effect between urban and rural groups.

Path	Rural (*n* = 6,916)	Urban (*n* = 2,711)	Δ*χ*^2^	*p*-value
Career expectation → Salary level	0.004	0.020	0.459	0.498
Partial moderation (Career expectation → Salary level)	0.020**	0.010	0.827	0.363
Salary level → Job satisfaction	0.079***	0.098***	1.351	0.245
Career expectation → Job satisfaction	0.121***	0.101***	1.263	0.261
Partial moderation (Career expectation → Job satisfaction)	−0.020***	−0.022**	0.049	0.824

*p < 0.1, **p < 0.05, ***p < 0.01.

#### 5.8.2 Gender heterogeneity analysis

Gender segregation in the labor market is a pronounced and persistent phenomenon. Studies indicate that, all other factors being equal, surveyed women’s income levels are approximately 60% of men’s income, and this disparity widens at higher income quantiles (He et al., 2020). In addition, significant gender differences exist regarding labor participation and occupational status, both of which influence women’s job satisfaction to varying degrees.

Using a multi-group structural equation model, this study assessed gender differences in path coefficients and mediation effects. Results revealed a significant gender difference in the relationship between career expectations and salary level (Δχ^2^ = 5.454, *p* < 0.05), with a significant positive correlation observed only for males. In contrast, no significant gender difference emerged in the association between salary level and job satisfaction (Δχ^2^ = 2.541, *p* > 0.05), with salary level significantly and similarly enhancing job satisfaction among both males and females. Additionally, a significant gender difference was observed in the correlation between career expectations and job satisfaction (Δχ^2^ = 1,077, *p* < 0.001), with males demonstrating a stronger correlation. The moderating effects on both paths, career expectations to salary level and career expectations to job satisfaction, showed no significant gender differences (Tables 11, 12).

**Table 11:** Heterogeneity of the mediation model between male and female groups.

Path	Male (*n* = 3,442)	Female (*n* = 6,185)	Δ*χ*^2^	*p*-value
Career expectation → Salary level	0.057***	0.010	5.454*	0.020
Salary level → Job satisfaction	0.084***	0.109***	2.541	0.111
Career expectation → Job satisfaction	0.263***	0.230***	1077***	<0.001

*p < 0.1, **p < 0.05, ***p < 0.01.

**Table 12:** Heterogeneity of the moderated mediation effect between male and female groups.

Path	Male (*n* = 3,442)	Female (*n* = 6,185)	Δ*χ*^2^	*p*-value
Career expectation → Salary level	0.033	−0.004	2.398	0.122
Partial moderation (Career expectation → Salary level)	0.013	0.025***	1.217	0.27
Salary level → Job satisfaction	0.072***	0.095***	2.452	0.117
Career expectation → Job satisfaction	0.123***	0.112***	0.478	0.489
Partial moderation (Career expectation → Job satisfaction)	−0.026***	−0.016**	1.629	0.202

*p < 0.1, **p < 0.05, ***p < 0.01.

From the above results, urban–rural differences primarily emerge in the relationship between career expectations and job satisfaction, with rural youth exhibiting a slightly stronger positive correlation than urban youth. Gender differences manifest primarily in the relationships between career expectations and salary levels and between career expectations and job satisfaction. Specifically, career expectations significantly and positively correlate with salary levels only for males, while this relationship is not significant for females. Additionally, males exhibit a slightly stronger positive correlation between career expectations and job satisfaction than females. Thus, these findings partially support Hypothesis 4.

## 6 Discussion

Taking G Province as a case study, this research constructs a core analytical framework based on expectancy theory, integrating SCCT and the EDM to explore multiple explanatory mechanisms linking career expectations and job satisfaction among youth in underdeveloped regions of China.

### 6.1 Theoretical contributions of this study

#### 6.1.1 Support, expansion, and boundary definition of SCCT

This study empirically confirms SCCT’s mediating pathway of “outcome expectation → objective reward → satisfaction” and proposes two significant theoretical expansions. First, it identifies structural constraints within underdeveloped regions as theoretical boundaries, reflected by the relatively weak mediating effect of salary level. This indicates that, in secondary labor markets characterized by a shortage of high-quality positions, the efficiency of converting the “outcome expectation → objective reward → satisfaction” pathway depends heavily on labor market structural conditions. Second, this research develops the traditional “satisfaction model” into a “moderated satisfaction generation model.” Previous studies typically positioned job preparation as a distal background variable preceding self-efficacy. However, this study finds that job preparation, as a behavioral manifestation of self-efficacy, plays a dual moderating role within the SCCT framework. This suggests that the conventional “human capital input–output” perspective is inadequate for explaining outcomes in underdeveloped regions: in secondary markets marked by an absolute shortage of high-quality jobs, adequate preparation not only enhances output efficiency but also systematically elevates reference benchmarks, producing novel outcomes beyond existing theoretical expectations.

#### 6.1.2 Empirical verification and enhancement of the EDM

The central premise of EDM is that satisfaction arises from the gap between actual experience and prior expectations. This study confirms a significant positive main effect of career expectations on satisfaction (H1), validating expectations as a reference benchmark. Two additional findings enhance the model empirically: First, the negative moderating effect of job preparation (H3b) indicates that adequate preparation raises individuals’ reference benchmarks and enables youth to develop more specific and clear expectations regarding job content. Consequently, when available positions in underdeveloped regions fail to match this elevated expectation, dissatisfaction resulting from this gap is greater compared to individuals who are less prepared. Second, this study reveals that the same individuals holding identical career expectations experience vastly different satisfaction outcomes depending on labor market structures, demonstrating that structurally diverse employment mechanisms affect even seemingly homogeneous youth cohorts.

#### 6.1.3 Verification and expansion of previous research frameworks on youth employment outcomes

Previous studies implicitly assumed that increased education, clearer career planning, and active job search preparation would linearly enhance employment quality. This study demonstrates that while job preparation does yield incremental improvements in salary, it does not sufficiently overcome structural constraints imposed by an absolute shortage of high-quality jobs, nor does it linearly enhance satisfaction as previously hypothesized. Recent literature on youth employment in developing countries similarly highlights an expectation-reality gap. This study further advances these findings by identifying job preparation as an “amplifier,” rather than a “mitigator,” of this gap.

### 6.2 Policy implications

Based on the above analysis and conclusions, the following policy implications are proposed. First, it is crucial to establish a hierarchical intervention approach. At the individual level, youth should be encouraged to obtain accurate labor market information through workplace visits, career dialogs, and practical work experience. These strategies will enable youth to develop “calibrated career expectations” and foster psychological resilience to mitigate dissatisfaction resulting from expectation gaps. At the policy level, governments, educational institutions, and employers should collaborate to form an integrated support system focused on “expectation calibration, skill empowerment, position expansion,” moving beyond traditional frameworks that emphasize preparation alone.

Second, four specific policy measures should be implemented: (1) Targeted career guidance: Employment services should integrate “realistic job preview” modules that transparently disclose salary ranges, career advancement timelines, and workload expectations, thereby reducing unrealistic benchmarks. (2) Wage support programs: Provide wage subsidies to enterprises hiring rural youth and women, thereby addressing structural disadvantages arising from weakened salary mediation channels. (3) Specialized employment service windows for rural youth: Establish dedicated service centers at county and township levels, supplemented by creating flexible-hour positions and facilitating urban–rural labor cooperation. (4) Integrated programs for pre-employment preparation and expectation management: Vocational training should include psychological resilience building and comprehensive employment information dissemination to address the paradox of diminishing satisfaction gains with increased preparation.

Third, align policies with regional equity and sustainable employment objectives. Underdeveloped regions should increase the total availability of high-quality positions through industrial upgrading and inter-regional labor cooperation, thereby enhancing labor market “structural carrying capacity.” Concurrently, policies tailored to urban–rural and gender differences should reduce job satisfaction disparities, strengthen youths’ intentions to remain employed locally, and foster a virtuous cycle of “skill adaptation, position expansion, retention willingness,” thus promoting high-quality, comprehensive regional employment.

### 6.3 Limitations and future research directions

This study employed cross-sectional survey data from G Province, China, using a moderated mediation model to explore complex relationships among career expectations, job preparation, salary levels, and job satisfaction among youth in underdeveloped regions. Nonetheless, several limitations exist. First, the cross-sectional and self-reported nature of the data necessitates cautious interpretation regarding causal inferences, as these measures may also be influenced by common method bias.

Second, although G Province exemplifies an underdeveloped region, findings from this area may not generalize universally to all underdeveloped contexts. Additionally, the research model omits variables such as family expectations, local industrial and employment structures, labor demand, employment contract stability, educational mismatch, and family socioeconomic status. Such omissions may introduce variable bias or reduce the explanatory power of the model. While these limitations do not invalidate the findings, they potentially affect the comprehensiveness of the explanations provided.

Third, the dependent variable “job satisfaction” was measured using a single item. Although methodological studies generally affirm the robustness of single-item measurements, and the study’s core conclusions have passed multiple rigorous robustness checks, this choice could still potentially influence parameter estimation accuracy.

In summary, future research can be expanded in the following directions: (1) include comparative samples from both developed and underdeveloped regions using a multi-wave longitudinal design (pre-graduation and post-graduation follow-up); (2) adopt multi-level modeling to examine cross-level moderation effects of macro-level variables, thereby enhancing causal inference and analyzing change trajectories; (3) incorporate additional mediating and moderating variables relevant to youth employment to enrich research insights; and (4) employ more precise variable design and rigorous structural equation modeling methods, supplemented by qualitative methodologies such as in-depth interviews and case studies, to deepen understanding of underlying mechanisms. Collectively, these steps can provide comprehensive theoretical support and practical guidance for optimizing youth employment support systems in underdeveloped regions.

## 7 Conclusion

### 7.1 Asymmetric patterns between salary mediation and direct effects of career expectations

Career expectations are significantly and positively correlated with job satisfaction, and salary level partially mediates this relationship. However, the indirect effect through salary accounts for only 1.14% of the total effect. This finding aligns with the conclusion by Judge et al. (2010), indicating limited correlation strength among objective salary, salary satisfaction, and job satisfaction. Thus, in underdeveloped regions, salary is not the dominant mechanism for enhancing job satisfaction; instead, the direct psychological benefit derived from career expectations is more influential.

### 7.2 The “double-edged sword” effect of job preparation

The study reveals an apparently paradoxical result: job preparation positively moderates the relationship between career expectations and salary levels, suggesting that better-prepared youth are more likely to translate expectations into higher salaries. Conversely, it negatively moderates the direct relationship between career expectations and job satisfaction, meaning increased preparation reduces the satisfaction derived from career expectations. Cavallari et al. (2024) note that labor markets in developing countries inherently lack sufficient absorption capacity for highly skilled workers, with initial employment typically concentrated in low-skilled secondary positions or informal sectors, lacking systematic channels for human capital returns. Under these structural conditions, the paradox can be explained by three theoretical pathways: First, heightened job preparation elevates reference standards, amplifying expectation-disconfirmation effects. Youth with greater preparation levels tend to compare actual employment outcomes against elevated standards developed during their preparation, intensifying feelings of unmet expectations and negatively moderating satisfaction. Second, psychological contract theory asserts that individuals hold beliefs regarding reciprocal obligations in employment relationships (Rousseau, 1989). Youth preparation in underdeveloped regions fosters high expectations about obligations employers should fulfill. When actual employment outcomes fail to meet these internalized obligations, psychological contract fulfillment is significantly lower than anticipated, thereby diminishing job satisfaction. Third, youth with higher preparation levels acquire extensive industry information, raising their standards for desirable jobs. Consequently, inflated expectations may accelerate satisfaction declines, resulting in diminishing marginal returns from job preparation.

### 7.3 Differentiation and convergence among different groups

The direct effect of career expectations on job satisfaction is significantly stronger for males compared to females, and for rural youth compared to urban youth, highlighting structural disparities in expectation conversion efficiency across groups. However, salary significantly enhances job satisfaction across all subgroups with similar magnitude, reinforcing salary’s universal role as a fundamental employment evaluation criterion. Additionally, the negative moderating effect of job preparation remains significant across all subgroups without notable group differences, suggesting the widespread applicability of the “preparation paradox” in underdeveloped regions.

## Data Availability

The raw data supporting the conclusions of this article will be made available by the authors, without undue reservation.
